# Comparison of antibiotic supplementation versus a yeast-based prebiotic on the cecal microbiome of commercial broilers

**DOI:** 10.1371/journal.pone.0182805

**Published:** 2017-08-24

**Authors:** Si Hong Park, Sang In Lee, Sun Ae Kim, Karen Christensen, Steven C. Ricke

**Affiliations:** 1 Center of Food Safety, Department of Food Science, University of Arkansas, Fayetteville, AR, United States of America; 2 Department of Poultry Science, University of Arkansas, Fayetteville, AR, United States of America; Hospital for Sick Children, CANADA

## Abstract

Prebiotics are defined as fermentable food ingredients that selectively stimulate beneficial bacteria in the lower gastrointestinal tract of the host. The purpose of this study was to assess growth performance of broilers and the cecal microbial populations of an antibiotic, BMD50, supplemented birds compared to broiler chickens fed the prebiotic, Biolex^®^ MB40. Weight response data including feed conversion ratios (FCR), carcasses without giblets (WOG), wing, skin, white meat were collected during processing. Extracted DNA from cecal contents was utilized for microbiome analysis via an Illumina Miseq. In conclusion, white meat yield of Biolex^®^ MB40 supplemented group exhibited significant improvement compared to both negative control (NC) and BMD50 supplemented groups. In addition, antibiotic significantly decreased level of *Lactobacillus* in 2 wk compared to other groups. A significantly higher percentage of *Campylobacter* was observed from the 4 wk old birds treated with antibiotic BMD50 compared to the NC and prebiotic group. Retention of broiler performance and improvement of white meat yield suggest that the prebiotic MB40 appears to be a potential alternative to replace the antibiotic growth promoter.

## Introduction

In 1995, Gibson and Roberfroid first defined prebiotics as non-digestible food ingredients that selectively promote beneficial bacteria such as *Bifidobacteria* and *Lactobacillus* [1] and ultimately enhance health of the host by altering the microbial populations in the gut. Prebiotics are becoming more attractive as alternative feed supplements in animal production because it has been suggested that common usage of antibiotics in agricultural production could result in increases of antibiotic resistant bacteria [2–8]. Prebiotics utilize various mechanisms to improve health of the host including short chain fatty acids (SCFAs) production, pH adjustment and competing for binding sites against pathogens [9–12]. For example, fructooligosaccharides (FOS) and galactooligosaccharides (GOS) are substrates for fermentation by *Bifidobacteria* and *Lactobacillus* thus, leading to increased SCFA production which in turn inhibits colonization and growth of pathogens [13–16].

The commercial prebiotic evaluated in this study, Biolex^®^ MB40 (Leiber GmbH, Hafenstraße, Germany), consists of 1,3–1,6- β-D-glucan and mannanoligosaccharides (MOS) which are derived from the cell walls of *Saccharomyces cerevisiae*, and several studies have shown their positive effects on growth performance of broilers [17, 18]. According to Hooge [17], supplementation of MOS exhibited statistically equivalent broiler body weights with antibiotic amendment groups while significant improvements in final body weight of broilers were observed compared to the negative control group. In addition, feed conversion ratio (FCR) significantly improved and mortalities were decreased in birds fed MOS diets by an average of 1.99 and 21.4% respectively compared to negative control groups but did not when compared to the antibiotic supplemented group. Hooge [17] reported that the greatest attribute of the MOS diet was the ability to decrease mortalities because it was the only attribute that was significantly different compared to the antibiotic diet fed group.

One of the distinct features to the MOS is the ability to bind to mannose-specific type-1 fimbriae of pathogen therefore, prevent colonization of pathogens [19]. Receptor competition against pathogens is mediated by high affinity ligands derived from the yeast cell wall [20]. One of the major foodborne pathogens, *Salmonella* utilizes this type-1 fimbriae thus, reduction of *Salmonella* concentration is expected upon introduction of MOS. Spring *et al*. [21] and Oyofo *et al*. [22, 23] observed *Salmonella* reduction in broilers by adding mannose to their diets. In a previous report Lee *et al*. [24] detected low levels of *Salmonella* from the same cecal samples used for the current study. In addition to improvement of growth performance and suppression of *Salmonella* colonization, supplementation of MOS also exhibited elevation of immunoglobulin A (IgA) and immunoglobulin G (IgG) level [25, 26].

The current study evaluated microbial populations of individual birds in each group using Next Generation Sequencing (NGS) approaches on samples collected from the study by Lee *et al*. [24] where polymerase chain reaction based denaturing gradient gel electrophoresis (PCR-DGGE) had been utilized for comparing cecal microbial populations. According to the previous report using a PCR-DGGE approach, prebiotic supplemented group exhibited very similar patterns with antibiotic supplemented groups prior to wk 2 but were similar to the negative control group as the birds became more mature. However, DGGE is limited for comprehensive identification of individual organisms in these complex microbial populations making it difficult to detect shifts in microbial groups comprising the range of taxonomic groupings. In-depthtaxonomic identification is now possible with next generation 16S rDNA microbiome sequencing and this approach has been shown to provide more information on cecal microbial populations when compared directly with DGGE analyses in previous studies in our laboratory [27]. Therefore as a followup analyses to our previous work [24], the objectives of the current study include not only bird performance assessment but Illumina Miseq 16S rDNA sequencing analysis of the cecal microbiome of conventionally raised broilers fed with commercial prebiotic MB40 compared to negative control group and antibiotic BMD50 fed birds.

## Materials and methods

### Broiler housing

**Fig 1:**
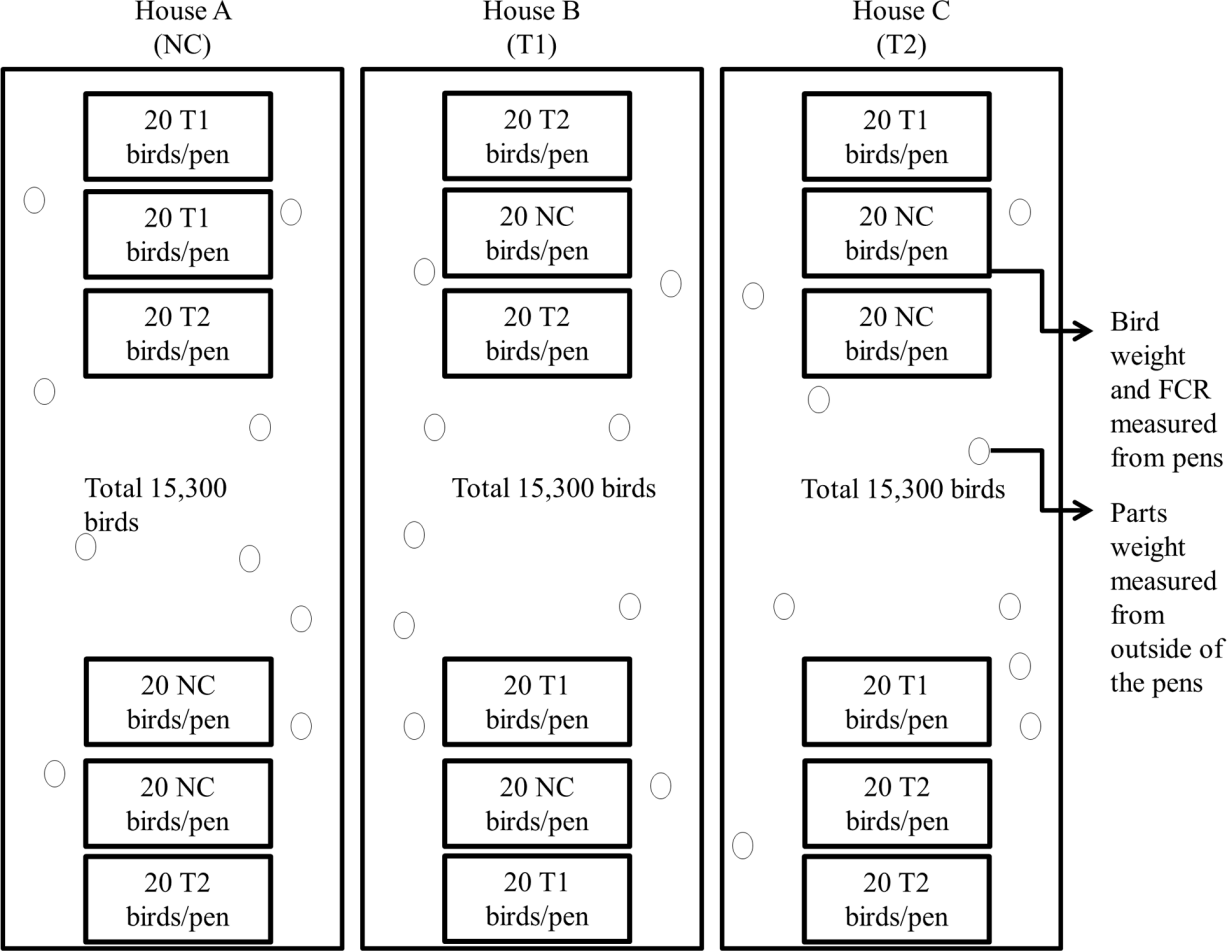
Experimental design of chicken housing. NC: Feed only; T1: 0.05% BMD 50; T2: 0.2% Biolex^®^ MB40.

Three houses were assigned for each treatment and each house contained 15,300 Hubbard x Cobb straight run broilers (OK Foods, Fort Smith, AR) (Fig 1). The birds were identified with the nametag of the corresponding treatment and house number when sampled to avoid confusion. In addition, three pens in two locations were set up within each house and twenty birds from each treatment group were randomly placed in one pen at each location to avoid a house effect (Fig 1). Diets for birds consisted of commercial starter, grower, finisher 1 and 2. The only difference between diets was that T1 group received 0.05% of BMD50 and T2 group consisted 0.2% of Biolex^®^ MB40. Since the birds were raised in an off-campus commercial farm, the current study was exempted from review by the University of Arkansas Institutional animal care and use committee (IACUC). No researchers at the University of Arkansas were involved directly in any aspects of the chicken growth part of the study. Therefore, authors did not need permission to access the farm since samples were collected by farm employees and subsequently transported to the laboratory at the University of Arkansas. In addition, the National chicken council (NCC) guidelines were (http://www.nationalchickencouncil.org) followed by the commercial cooperators to ensure internal animal welfare. Ten birds from each treatment were chosen for sampling of cecal contents as described previously [24].

### Broiler performance

The twenty birds that resided in the respective pens were weighed at 14, 28, 40 and 53 days of age and recorded. In addition, carcasses without giblets (WOG), wing, skin breast and tender weights were measured from randomly selected 100 birds (50 males and 50 females) that were located in the house but outside the pens at 53 days of age. Birds that lost tags or were condemned by the USDA inspector were exempted from statistical analysis. Finally feed conversion ratios (FCR) were determined by feed intake and body weight at 28, 40 and 53 days of age. White meat yields were calculated by dividing sum of breast and tenders by live weight of randomly selected 100 birds within each house. Recorded data were imported by Microsoft Excel and JMP^®^ Genomics for analysis of variance (ANOVA). *P*-values less than 0.05 were interpreted as significant differences among treatments.

### Cecal microbial population assessment

Cecal contents (200 mg) from each birds were collected for DNA isolation utilizing QIAamp DNA Stool Mini Kit (Qiagen, Valencia, CA, US). The concentration of extracted DNA was diluted to 10 ng μL^-1^ for the preparation of a sequencing library targeting the V4 region of 16S rRNA [28]. Based on the recommendation of the manufacturer's protocol, isolated DNA samples were amplified and normalized using dual-index primers and SequalPrep^TM^ Normalization kit (Life Technology, Carlsbad, CA, US). The library was constructed by combining 5 μL of each normalized aliquot samples for further assessment. Library concentration and product size were confirmed using a KAPA Library Quantification Kit (Kapa Biosystems, Woburn, MA, US) via a quantitative PCR (qPCR, Eppendorf, Westbury, NY, US) and an Agilent 2100 Bioanalyzer system (Agilent, Santa Clara, CA, US). The 20 nM of pooled library aliquot and the 20 nM of PhiX control v3 were combined with 0.2 N fresh NaOH and HT1 buffer and mixed a second time with 5% of the PhiX control v3. The 600 μL of the mixture containing pooled library aliquote, PhiX control v3, NaOH and HT1 buffer was subsequently loaded onto a MiSeq v2 Reagent cartridge to conduct sequencing.

### Microbiome sequencing analysis by QIIME pipeline

Sequencing read files were processed using Quantitative Insights into Microbial Ecology (QIIME) pipeline (version 1.9.0) [29]. Each of the operational taxonomic units (OTUs) was assigned to specific microorganisms to determine taxonomic levels and subjected to alpha and beta diversity analyses and tables were constructed by clustering sequences with 97% or higher identity based on Greengenes 16S rRNA gene database. In addition, OTUs that were not observed at least twice were excluded manually to eliminate possible erroneous reads from sequencing. Chimeras considered as sequences generated by multiple templates or parent sequences were identified and filtered by ChimeraSlayer script that utilizes BLAST. Also, the OTU table was subsampled or rarefied using a minimal observed OTU value to discard any samples that contained unusually fewer sequences. Subsequently, OTUs tables were converted to taxonomic tables for further analysis. Weighted and unweighted version of UniFRac graphs and rarefaction plots were generated for beta and alpha diversity tests, respectively. Taxonomic level data acquired by QIIME was imported by Microsoft Excel and JMP^®^ Genomics for ANOVA and a *P*-value of 0.05 to determine significant differences.

## Results and discussion

The current study is the continued analysis of previously published research of Biolex® MB40 by Lee *et al*. [24] where fingerprinting of cecal microbiota were analyzed by a PCR-DGGE. The current study focused on identification of the microbial population at the molecular taxonomic levels along with the growth and processing performance of the birds.

### Broiler performance

Growth performance including weight of the birds, FCR, weight of the parts and white meat yields of broilers are considered important since they directly relate to the market value of the bird [30, 31]. Average body weight and FCR are shown in Table 1. There were no significant differences among groups in terms of parts yield and FCR. These results agree with earlier studies by Waldroup *et al*. [32] and Midilli *et al*. [33] where no improvement of body weight and body weight gains were observed upon introduction of a MOS and yeast derived prebiotic. No significant improvement of FCR by prebiotic was also observed by Ignacio [34] however, FCR of broilers were significantly increased in the study by Midilli *et al*. [33] which agrees with observations by Sahane [35] and Pelícia *et al*. [36] when broilers were supplemented with MOS or a mixture of probiotic and prebiotic. In addition, the study by Biggs *et al*. [37] reported that MOS did not exhibit any effect on metabolizable energy (ME) but reduced amino acid digestibility of broilers until day 7 of age when MOS, inulin, oligofructose, short-chain fructooligosaccharide (SCFOS), and transgalactooligosaccharide were compared. In addition, Pelicano *et al*. [38] observed better weight gain and increased FCR when MOS was supplemented however, positive effects in growth performance were only observed when amendments were introduced at 21 days of age. Pelicano *et al*. [38] speculated that absence of better weight gains at 35 and 42 days of age may be due to the level of stress and dilution effect by other compounds in the grains such as non-starch polysaccharides and non-digestible oligosaccharides. Midilli *et al*. [33] and Roshanfekr and Mamooee [39] have hypothesized that the reason for inconsistent results among studies could be the differences in management, environmental conditions, stress and presence of unfavorable microorganism.

**Table 1:** Broiler body weight and FCR responses over ages and treatments.

		
	Age (Day)	NC (Feed only)	T1 (BMD50)	T2 (MB40)
Bird weight (kg)	14	0.68 ± 0.01	0.68 ± 0.00	0.68 ± 0.00
	28	1.60 ± 0.01	1.56 ± 0.02	1.57 ± 0.01
	40	2.65 ± 0.01	2.61 ± 0.02	2.64 ± 0.02
	53	3.71 ± 0.03	3.70 ± 0.07	3.85 ± 0.07
FCR	28	1.51 ± 0.01	1.53 ± 0.02	1.56 ± 0.03
	40	1.80 ± 0.01	1.81 ± 0.01	1.90 ± 0.07
	53	1.74 ± 0.03	1.66 ± 0.02	1.74 ± 0.04
	53(Mort Adj^1^)	1.69 ± 0.01	1.66 ± 0.02	1.68 ± 0.02

^1^Mort Adj includes total mortality weight in the FCR calculation

Another study by Park *et al*. [40], on the same prebiotic used in the current study, Biolex^®^ MB40, observed similar results of pasture flock broiler performance with no significant differences occurring in FCR and average bird body weights among treatment groups. However, the prebiotic MB40 results did match the responses seen with antibiotic fed birds therefore, prebiotic MB40 potentially could replace the benefit seen with the antibiotic supplement. Significant weight gains of the birds were observed in the study by Roshanfekr and Mamooee [39], when supplementation of MOS, Primalac (commercial probiotic) and a mixture of both increased FCR and average weight of the birds by 81.3, 73.5 and 148.8 g compared to the control group.

When comparing poultry processing responses including chicken part weights and white meat yield, only the white meat yield was significantly increased by supplementation of prebiotic MB40 compared to negative control group and antibiotic treated group (Table 2). According to Stevens [30], white meats are the most economically valuable part of broilers raised and processed in Europe. The white meat or the breast meat is particularly important to producers because studies have shown a high genetic correlation between white meat and the body weight of the birds [41]. Roshanfekr and Mamooee [39] observed significantly higher breast meat yield when the probiotic was supplemented however, the prebiotic supplemented group did not exhibit any improvement of breast meat yield.

**Table 2:** Comparison of chicken carcass parts yield as a percentage.

			Treatments	
Parts (kg)		NC (Feed only) n = 91	T1 (BMD50) n = 96	T2 (MB40) n = 97
	WOG	3.24 ± 0.39	3.34 ± 0.34	3.34 ± 0.41
	Wing	0.32 ± 0.05	0.32 ± 0.05	0.32 ± 0.05
	Skin	0.15 ± 0.03	0.15 ± 0.02	0.15 ± 0.03
	Breast	0.73 ± 0.10	0.75 ± 0.09	0.78 ± 0.10
	Tender	0.15 ± 0.02	0.15 ± 0.01	0.16 ± 0.02
		NC (Feed only)	T1 (BMD50)	T2 (MB40)
White meat yield (%)		24.36 ± 0.18^b^	24.35 ± 0.17^b^	25.15 ± 0.17^a^

WOG: Without giblets

Mean values in the same row followed by different superscript letters represent statistically significant differences (*P* < 0.05)

### Taxonomy summary

**Fig 2:**
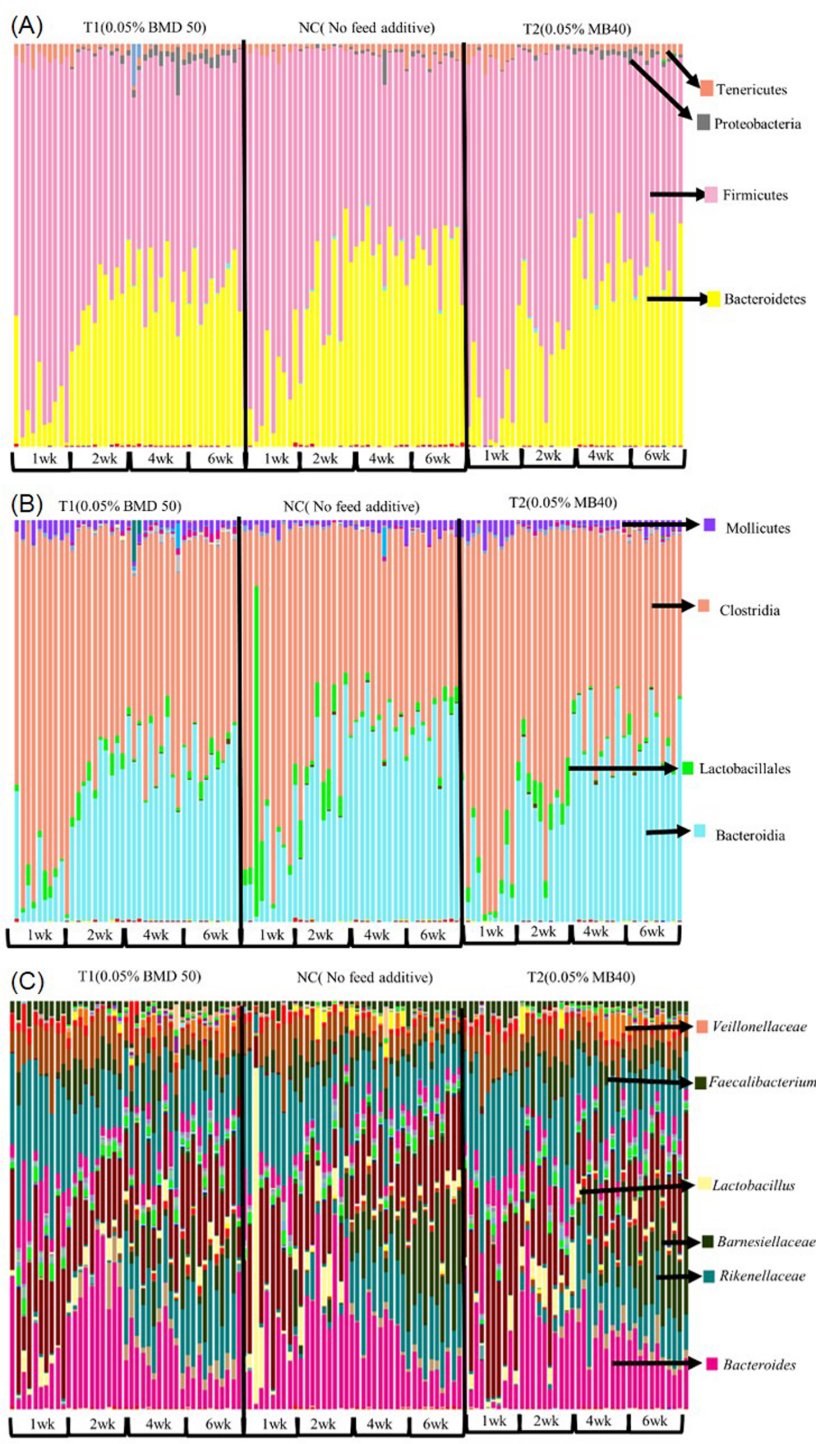
Taxonomy bar graphs of respective microorganisms in each taxonomy level generated by QIIME. A: Phylum level; B: Class level; C: Genus level.

Table 3 represents the total number of reads acquired by Illumina MiSeq and observation OTU count summary by the QIIME pipeline. The QIIME pipeline was able to identify and differentiate the 16S rRNA fragment of bacteria from phylum level to species level and taxonomy bar graphs could be generated for each bacterial grouping from each sample. According to QIIME analysis, the most abundant bacteria at the phylum level was Firmicutes with an average of 62.6%, followed by Bacteroidetes with an average of 33.7% (Fig 2A). Proteobacteria and Tenericutes were also detected with averages of 1 and 2.2% from the samples respectively (Fig 2A). Clostridiales were the most abundant microorganisms in the Firmicutes group, and Bacteroidia were commonly identified within the Bacteroidetes group. (Fig 2B). In addition, 3.4% of the Firmicutes were identified as Lactobacillales (Fig 2B). Proteobacteria and Tenericutes were divided into more specific sets of organisms at the order level consisting primarily of Enterobacteriales and Mollicutes (Fig 2B). While the sum of Bacteroidales and Clostridiales comprised more than 90% of the total microbial population, Bacteroidales were found predominantly in birds that were more or equal to 2 wk of age despite the treatment. The level of Bifidobacteriaceae was low and was detected in very few samples, thus a statistical comparison was not possible. Genus level graphs are shown in Fig 2C.

**Table 3:** Sequence reads number and BIOM table summary.

BIOM table (observation counts)				
Total observation	Min*	Max*	Median*	Mean*
10,030,751	42,204	134,689	82,611	83,589

*Counts per Samples

When prebiotics were first introduced, the only microorganisms that were noted as beneficial bacteria were *Lactobacilli* and *Bifidobacteria* [42]. However, more detailed investigations using various molecular techniques such as qPCR or NGS have revealed more complex outcomes when specific prebiotics are introduced to the host. Numerous studies have reported that specific prebiotic supplementation not only promotes *Lactobacilli* and *Bifidobacteria* but also improves glucose homeostasis, leptin sensitivity and intestinal homeostasis [43, 44]. In addition studies have suggested that prebiotic increases other beneficial microorganisms besides *Lactobacilli* and *Bifidobacteria* such as *Faecalibacterium prausnitzii* and *Akkermansia muciniphila* which in turn benefits the host [45–48].

### Microbial population shifts

According to Lee *et al*. [24], antibiotic, BMD 50 and prebiotic, MB40 treated groups exhibited greater similarities in early stages of bird growth based on the phylogenetic trees generated from analysis of band patterns produced by DGGE. Based on QIIME analysis, most of the bacteria did not exhibit significant differences in yield among different treatment groups except *Barnesiellaceae* which is a microorganism that commonly occurred in all groups at wk 4 according to previous DGGE-band sequencing analysis [24]. However, in the current study using NGS bacterial composition varied significantly by age of the birds. Firmicutes at the phylum level and Clostridiales at the order level decreased as birds became older with no significant differences between treatments (Table 4). Previously, DGGE-band sequencing also detected Clostridiales in all treatment groups [24]. Out of a total population of microorganisms, Firmicutes and Bacteroidetes constituted more than 90 percent of the population and this is consistent with the fact that Firmicutes and Bacteroidetes are the most abundantly found microorganisms and are associated with energy resorption rate in the gut [49–50]. Bacteroidales however, increased significantly each week until 4 wk and remained at a consistent level until 6 wk with no significant improvements among treatments unlike the previous DGGE-band sequencing analysis [24] which detected *Bacteroides dorei* and *Bacteriodes rodentium* only in the negative control group for wk 2 and 4 and were less frequent by 6 wk. Mollicutes levels were low in 2 and 4 wk but significantly higher levels were detected in 1 and 6 wk. However, the level of *Bacteroidaceae* increased significantly in 2 wk and decreased afterwards.

**Table 4:** Relative abundance of bacteria in phylum level (%).

	Relative abundance			
Organisms	1 wk	2 wk	4 wk	6 wk
Firmicutes	83.15 ± 1.69^a^	64.82 ± 1.66^b^	50.29 ± 1.66^c^	51.68 ± 1.66^c^
Clostridiales	77.06 ± 1.94^a^	58.29 ± 1.94^b^	48.88 ± 1.94^c^	49.37 ± 1.94^c^
Bacteroidales	11.47 ± 1.72^c^	31.91 ± 1.72^b^	43.98 ± 1.72^a^	43.47 ± 1.72^a^
Molicutes	3.16 ± 0.23^a^	1.35 ± 0.23^b^	01.41± 0.23^b^	2.51 ± 0.23^a^
Bacteroidacceae	11.44 ± 1.46^c^	26.77 ± 1.46^a^	17.84 ± 1.46^b^	9.60 ± 1.46^c^

Mean values in the same row followed by different superscript letters represent statistically significant differences (*P* < 0.05)

While both methods, DGGE-band sequencing and NGS results were somewhat similar there were differences as well. Such differences in outcomes between DGGE profiles and NGS microbiome sequencing of broiler cecal samples has been observed by our group in previous work [27]. Therefore, direct comparisons between the two methods must be exercised with caution as they differ considerably in both method and approach as discussed previously by Park et al., 2017 [27]. Obviously, DGGE-band sequencing relies on visualization of bands and physically excising these bands for sequencing which immediately introduces some operator error. In addition, DGGE-band sequencing [24] was based on hypervariable region 3 (V3) while the microbiome sequencing in the current study was based on V4. Both V regions have designated as optimal for these respective methods but the taxonomic outcome may be different as each has some inherent bias towards and against certain organisms [51]. Yu and Morrison (2004) [52] compared V regions of *rss* genes and concluded that V3 yielded the best DGGE profiles. For microbiome sequencing, generally V3-V4 or V4 are preferred due the coverage of both bacteria and archaea found in gut and fecal samples [51, 53–55]. However, after comparing the sequences of the V1-V8 regions from 110 different bacterial species Chakravorty et al. (2007) [56] observed that each V region yielded different levels of discrimination for different groups of organisms with certain organisms being more or less differentiated depending on the V region.

**Fig 3:**
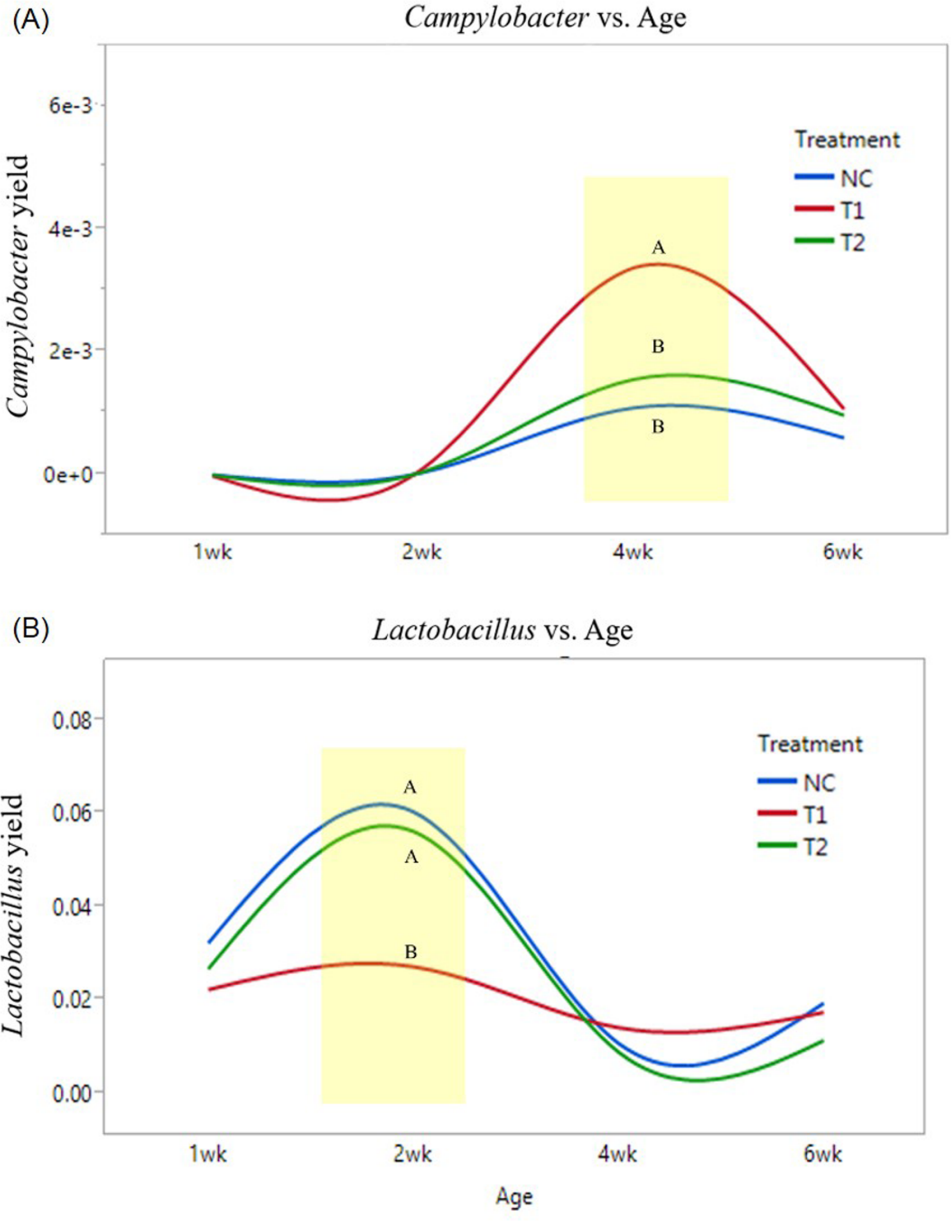
Relative abundance of *Campylobacter* and *Lactobacillus* by treatment and age of the birds (NC: Feed only; T1: 0.05% BMD 50; T2: 0.2% Biolex^®^ MB40). A: Comparison of *Campylobacter* yield by bird age and treatment; B: Comparison of *Lactobacillus* yield by bird age and treatment. (Different letters indicate significant differences, *P* value < 0.05)

According to QIIME, 67 of 120 samples contained *Campylobacter* however, 90% of *Campylobacter* were found in birds with 4 and 6 wk of age which indicates *Campylobacter* colonizes within birds in their later phases of growth. Recovery of *Campylobacter* among all microorganisms were averaged 0.3%. Several studies have shown that colonization of *Campylobacter* can be suppressed by introduction of *Lactobacillus* and *Bifidobacteria* [57–60] and a previous study by Kaakoush *et al*. [61] demonstrated a negative correlation between *Campylobacter* and *Lactobacillus* however, no statistical relationship was found in the current study where a significantly higher level of *Campylobacter* was observed at 4 wk in the antibiotic treated group compared to the negative control and prebiotic treated group (Fig 3A).

One of the major bacteria considered to be beneficial in broilers, *Lactobacillus* [62–63].was identified among all samples with an average of 3%. Levels of *Lactobacillus* observed in antibiotic treated groups were significantly lower compared to negative control and prebiotic treated groups at 2 wk (Fig 3B). In addition, prebiotic MB40 fed birds were the only group that exhibited significantly higher levels of *Lactobacilli* in 2 wk however, statistical differences did not occur after 2 wk.

### Alpha and beta diversity analysis

**Fig 4:**
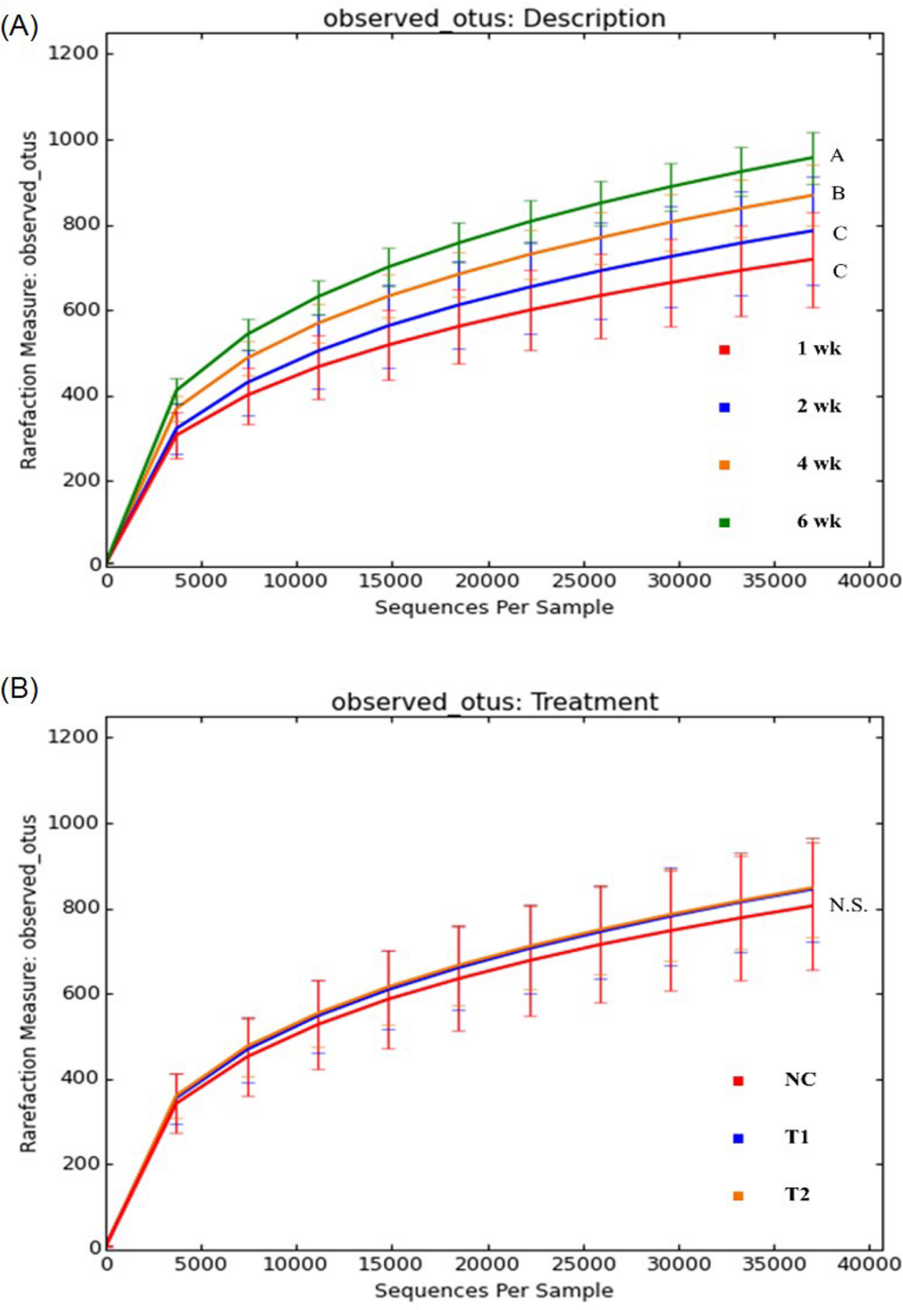
Rarefaction plots indicating amount of diversity by treatment (NC: Feed only; T1: 0.05% BMD 50; T2: 0.2% Biolex^®^ MB40). A: Rarefaction plot by bird age; B: Rarefaction plot by treatments. (Different letters indicate significant differences, *P* value < 0.05, N.S. stands for no significant difference)

Alpha diversity analysis were carried out by the QIIME pipeline to assess microbial diversity within each sample. Fig 4A and 4B represent rare classes detected for each of the sequences obtained from the samples based on observed OTUs metric system, which is commonly used for assessment of organism diversity. A rarefaction graph based on age (Fig 4A) demonstrated that birds develop a more complex population of microorganisms as they become older. A rarefaction graphs based on treatment (Fig 4B) revealed that prebiotic and antibiotic treated groups occurred more closely together compared to the NC group and a higher rarefaction was observed which indicates prebiotic or AGP amendments supported a more complex microbial diversity compared to the no feed amendment group. High similarity of microbial diversity between antibiotic, T1 and prebiotic, T2 groups agreed with the previous research result by Lee *et al*. [24] where the diversity of microbial populations had been assessed by PCR-DGGE.

**Fig 5:**
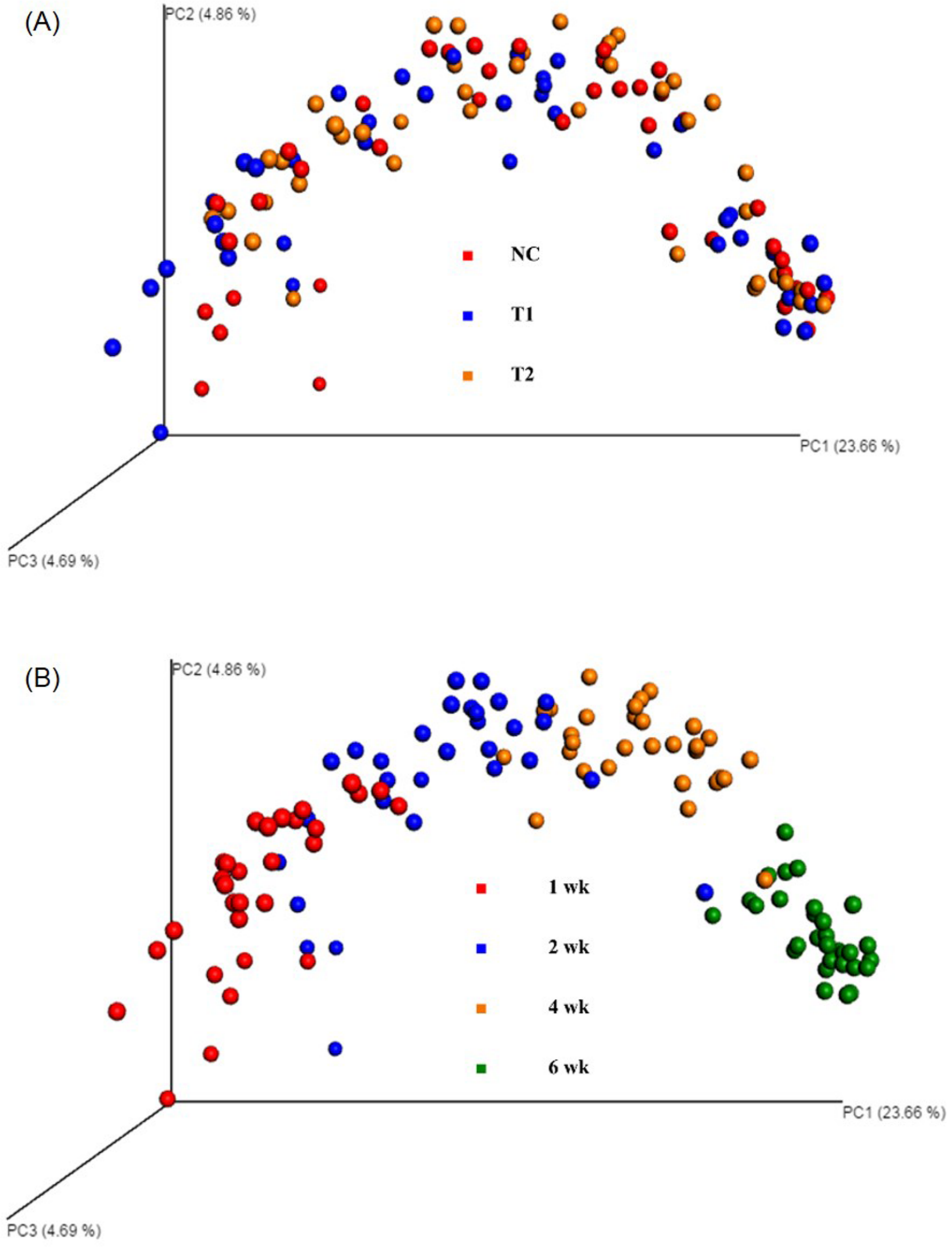
PCoA plots based on treatments and age of broilers (NC: Feed only; T1: 0.05% BMD 50; T2: 0.2% Biolex^®^ MB40). A: PCoA plot by treatments; B: PCoA plot by bird age.

Fig 5A and 5B represent PCoA beta diversity plots. PCoA plots based on treatment (Fig 5A) did not reveal any clustering patterns resulting in an ANOSIM R-value of 0.03 however, PCoA plot based on age (Fig 5B) exhibited considerable clustering with an ANOSIM R-value of 0.6 by age of the birds which implies maturity of the bird having a greater impact and evolving into a more uniform diversity of microorganisms regardless of the treatments used in the current study. In addition, clustering by samples originating from younger birds were more widely scattered than older birds, indicating a potential stabilization of the cecal microbiome as the birds mature. The observation of complex but highly similar diversity pattern in older birds agrees with previous studies where DGGE exhibited similar patterns as birds reached their marketing age [24].

## Conclusions

No statistical differences were detected in FCR or parts yield which leads to the conclusion that there were no variation in terms of chicken performance when BMD50 (T1) and MB40 (T2) were compared. Consistency of body weight, FCR and parts yields among prebiotic, antiobiotic and control groups was also observed in previous studies [64–65]. The growth performance between treatment and negative control groups were not statistically different except for the white meat yield. Growth performance evaluation of Hubbard x Cobb species fed with common corn-soybean meal by Dozier and Gehring (2014) [66] reported that body weight and FCR of the broilers were near 1.68 and 0.67 kg (1.59 and 0.68 kg in the current study) respectively at 28 days of age which suggest the housing and management system of the current study led to a maximum output of bird performance thus, no significant differences were observed.

Overall, commercial prebiotic, MB40 significantly improved white meat yield in conventionally raised broilers but was not accompanied by significant enhancement in other aspects including body weight, FCR and parts weight. According to the NGS approach to microbiome analysis a significant decrease in *Lactobacillus* level occurred in birds receiving antibiotic BMD50 supplementation at 2 wk. No significant reduction of chicken performance was observed thus, MB40 could be a viable alternative of in-feed antibiotic supplementation when addressing concerns related to the presence of antibiotic resistant bacteria.
